# Supplementary figures and images for: A novel mouse model for LAMA2-related muscular dystrophy with analysis of molecular pathogenesis and clinical phenotype
Source: eLife. 2025 Sep 17;13:RP94288. doi: 10.7554/eLife.94288 (PMC12443477; doi:10.7554/eLife.94288)

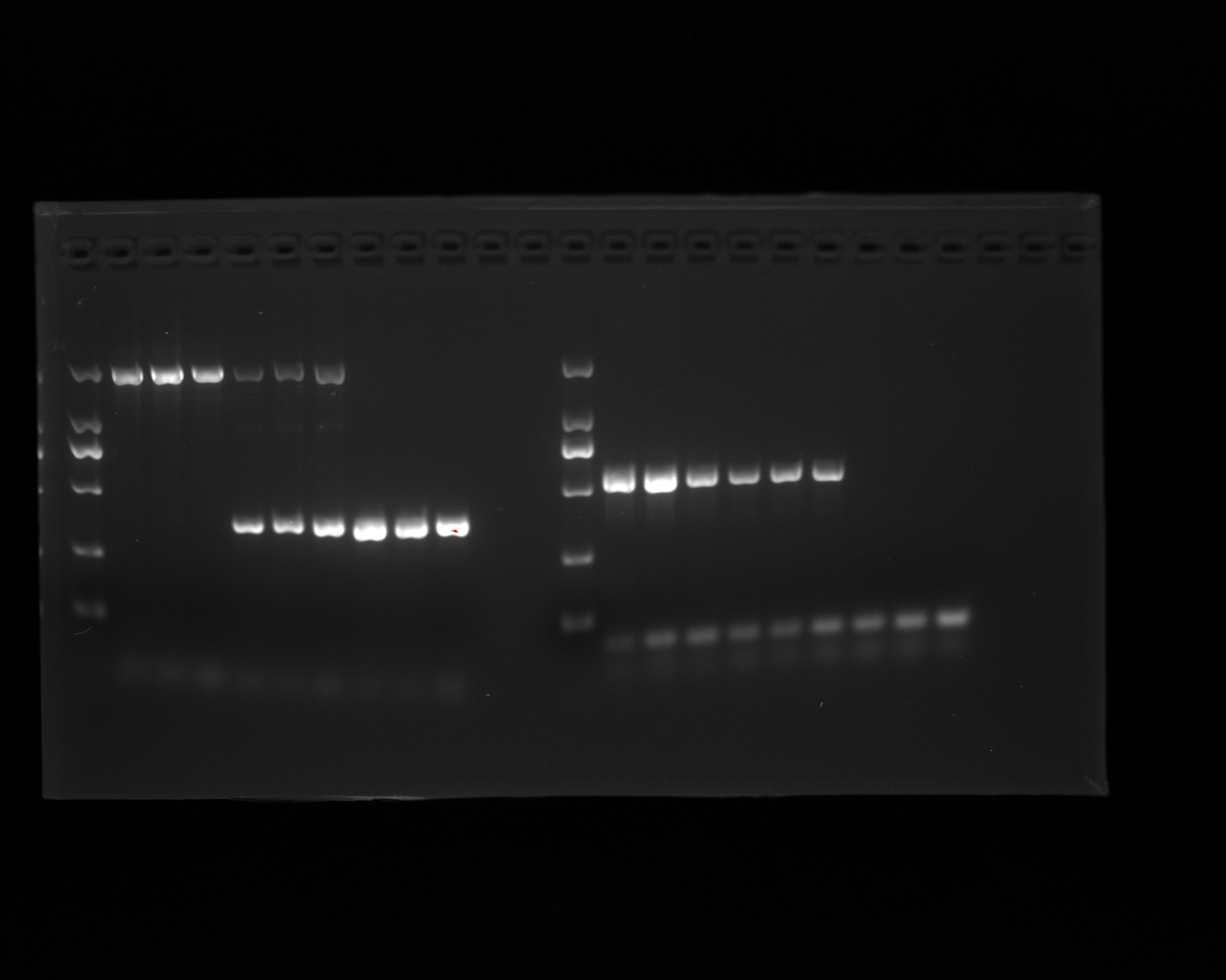

Supplement: Figure 1—source data 1. [file elife-94288-fig1-data1.zip › Figure 1–source data 1/PCR analysis for genotype identification.jpg]

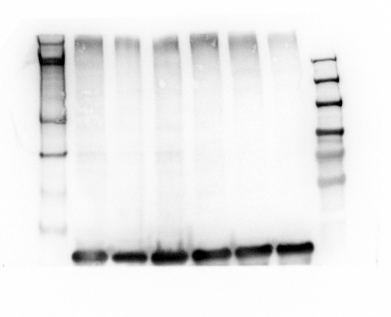

Supplement: Figure 4—source data 1. [file elife-94288-fig4-data1.zip › Figure 4–source data 1/GAPDH.tif]

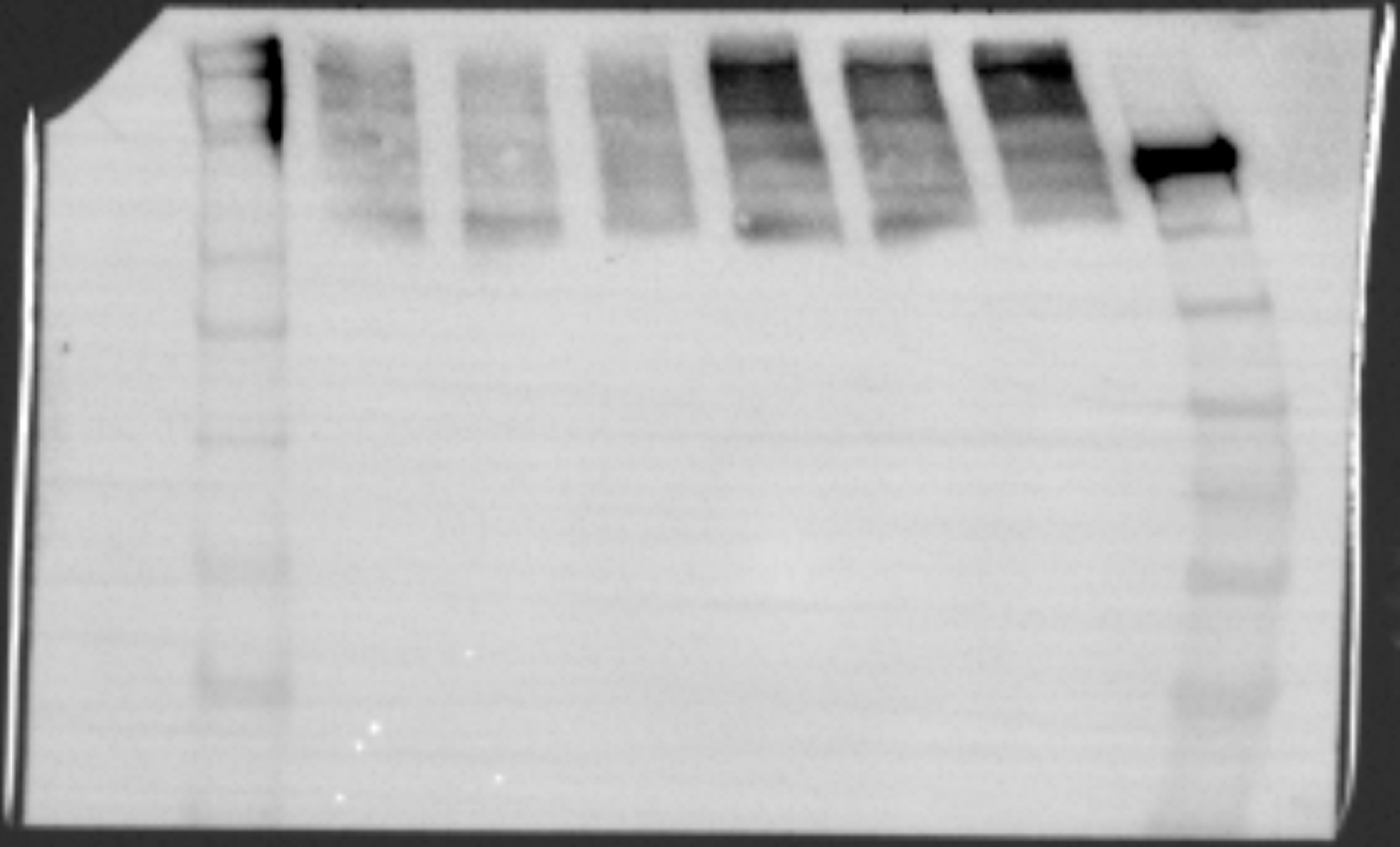

Supplement: Figure 4—source data 1. [file elife-94288-fig4-data1.zip › Figure 4–source data 1/Laminin a2.tif]

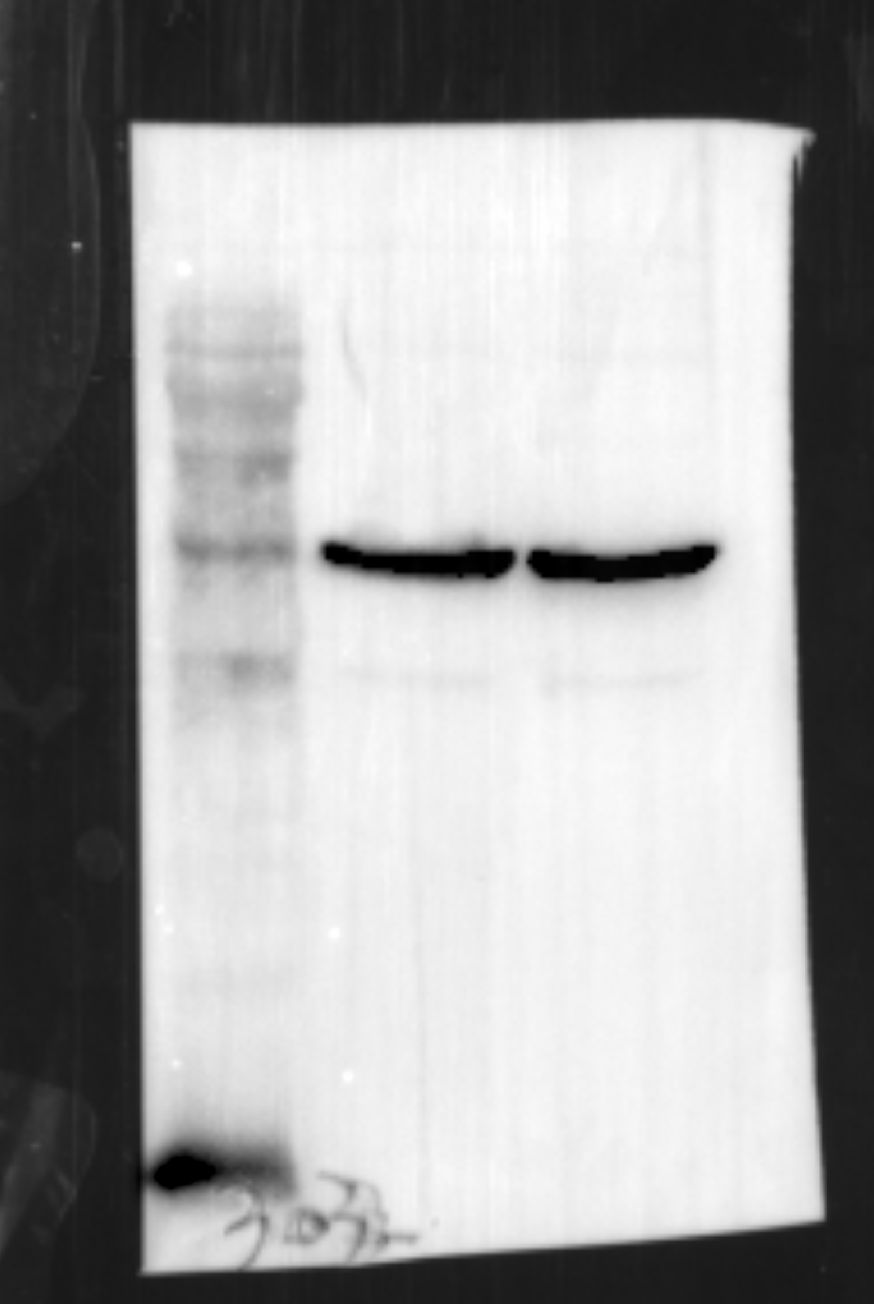

Supplement: Figure 8—source data 1. [file elife-94288-fig8-data1.zip › Figure 8–source data 1/a-actin.tif]

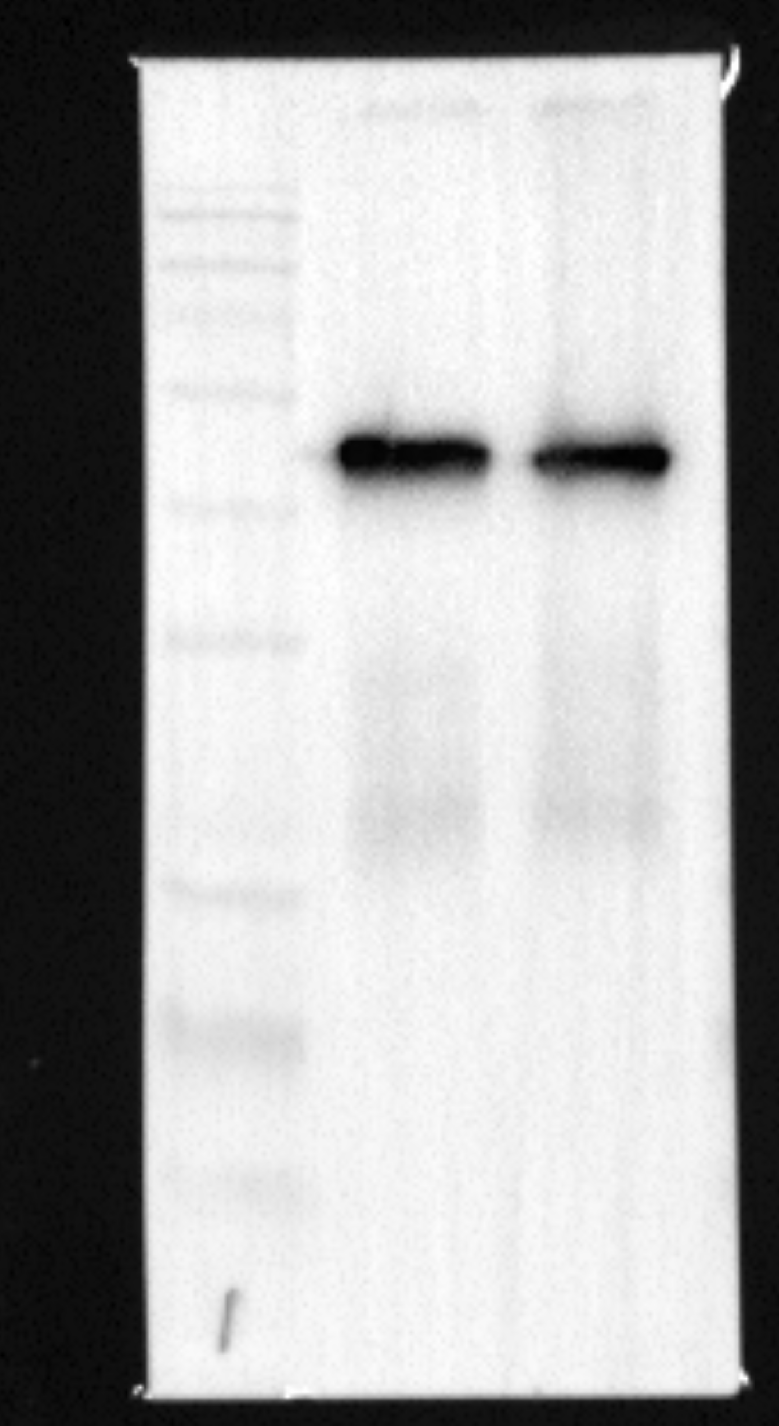

Supplement: Figure 8—source data 1. [file elife-94288-fig8-data1.zip › Figure 8–source data 1/b-tubulin.tif]

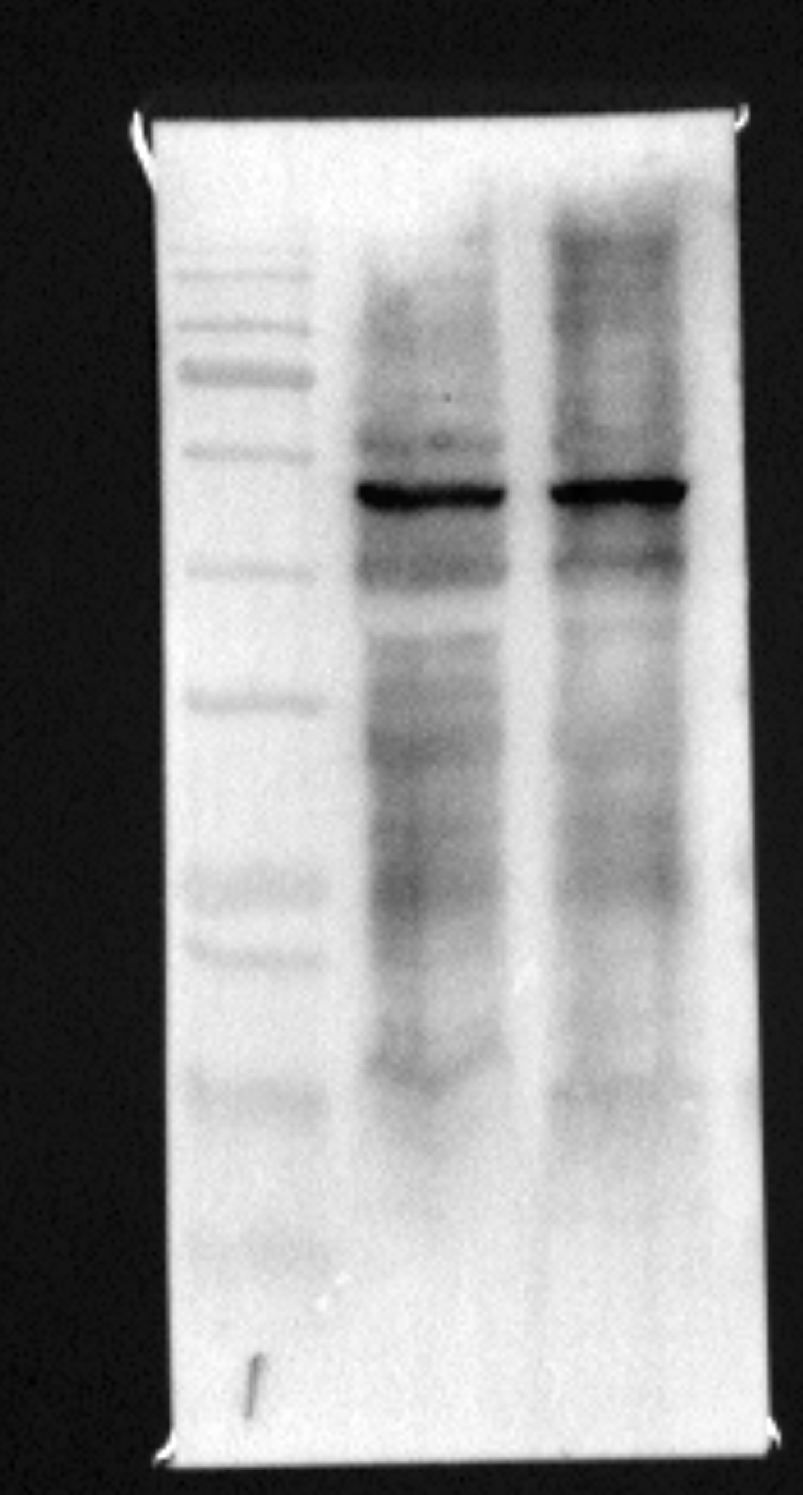

Supplement: Figure 8—source data 1. [file elife-94288-fig8-data1.zip › Figure 8–source data 1/Desmin.tif]

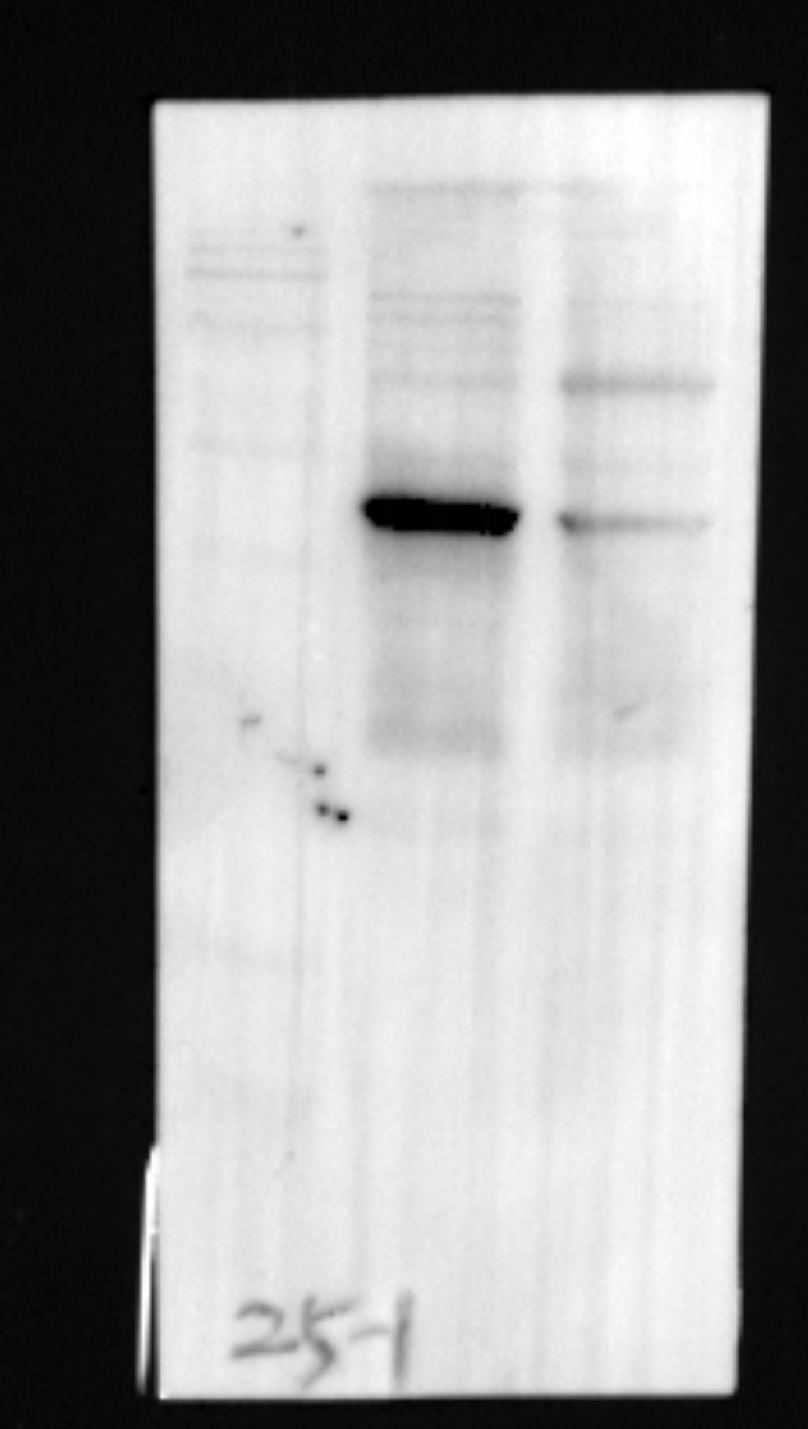

Supplement: Figure 8—source data 1. [file elife-94288-fig8-data1.zip › Figure 8–source data 1/F-actin.tif]

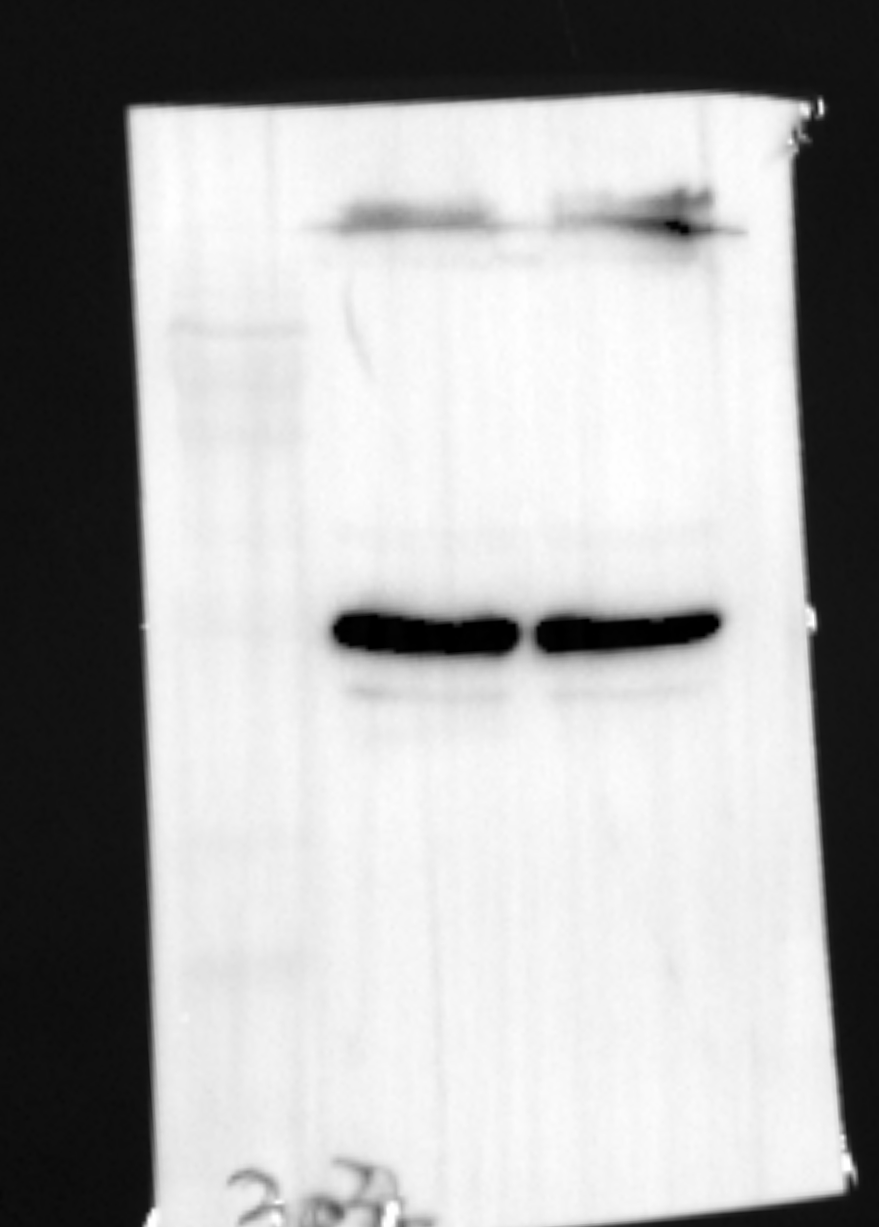

Supplement: Figure 8—source data 1. [file elife-94288-fig8-data1.zip › Figure 8–source data 1/GAPDH (a-actin).tif]

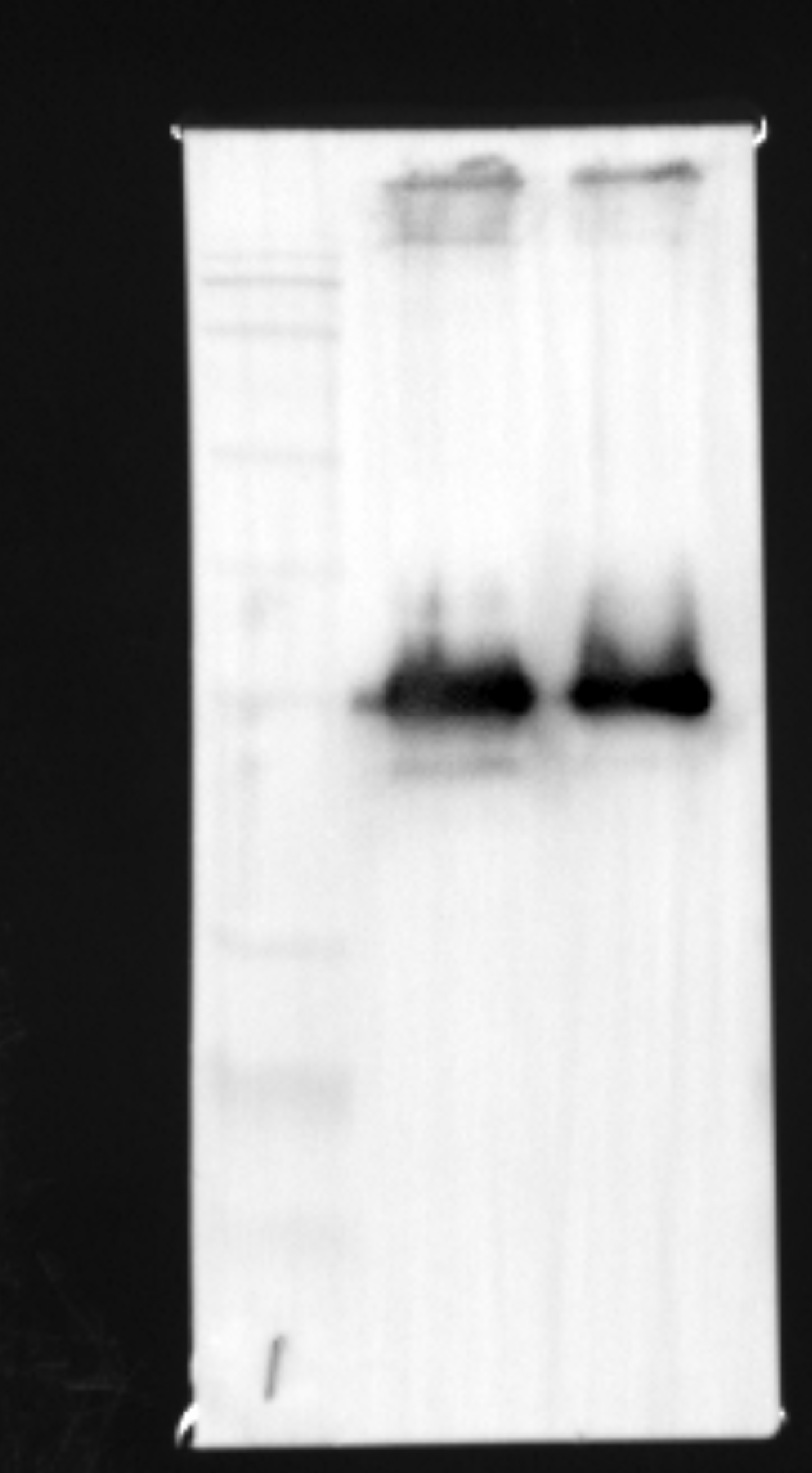

Supplement: Figure 8—source data 1. [file elife-94288-fig8-data1.zip › Figure 8–source data 1/GAPDH (Desmin, b-tubulin).tif]

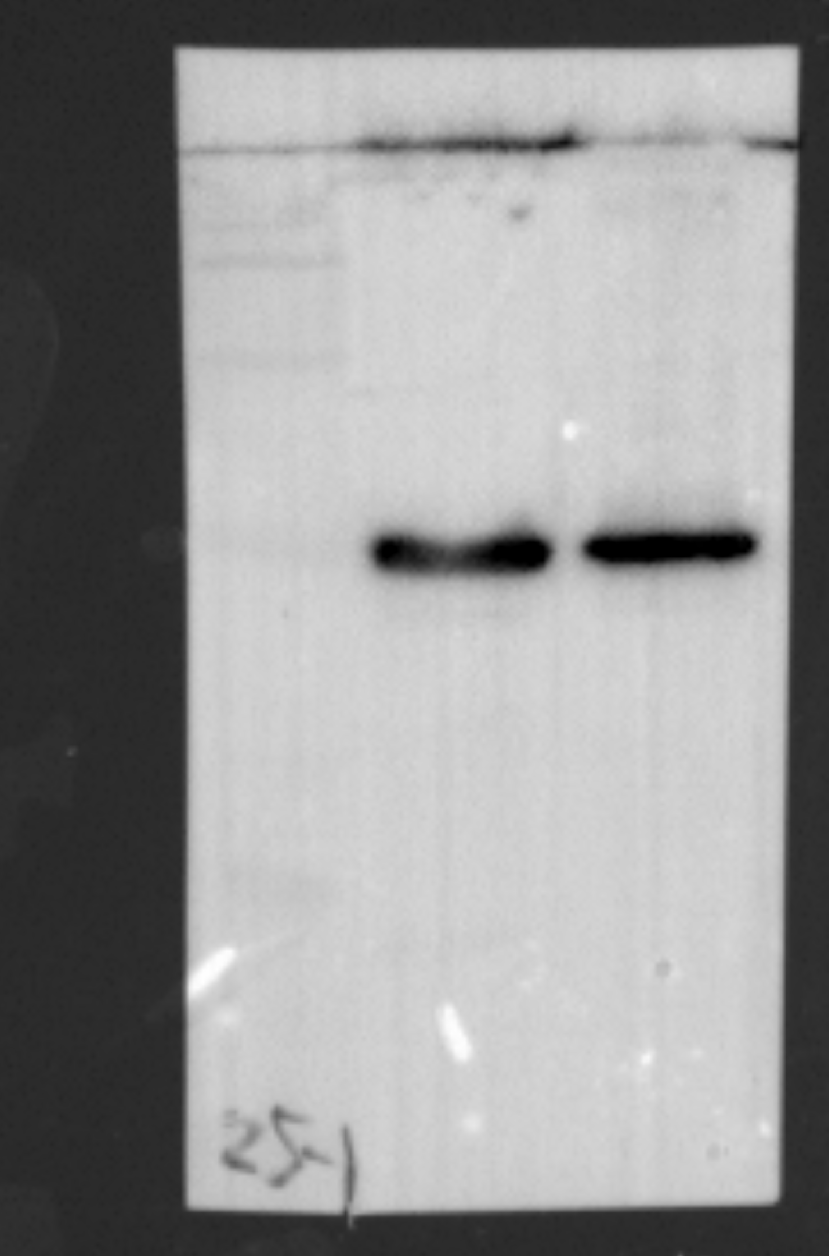

Supplement: Figure 8—source data 1. [file elife-94288-fig8-data1.zip › Figure 8–source data 1/GAPDH (MYH2).tif]

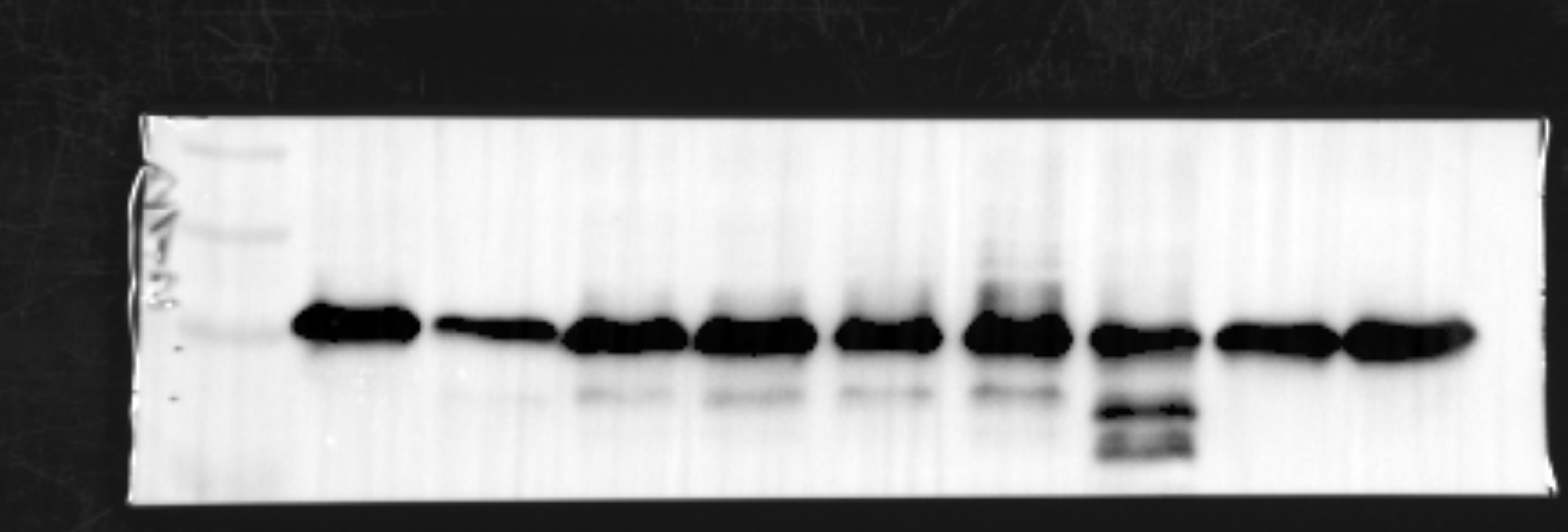

Supplement: Figure 8—source data 1. [file elife-94288-fig8-data1.zip › Figure 8–source data 1/GAPDH (MYOD1).tif]

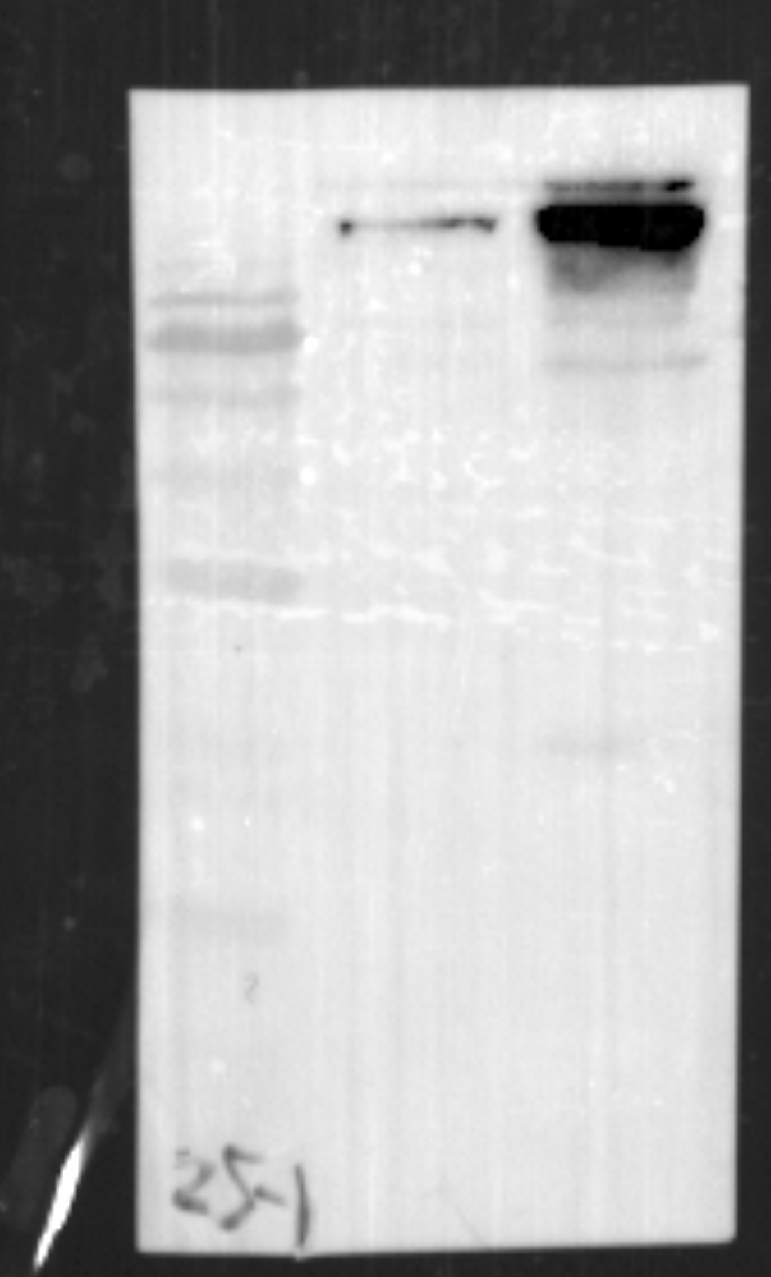

Supplement: Figure 8—source data 1. [file elife-94288-fig8-data1.zip › Figure 8–source data 1/MYH2.tif]

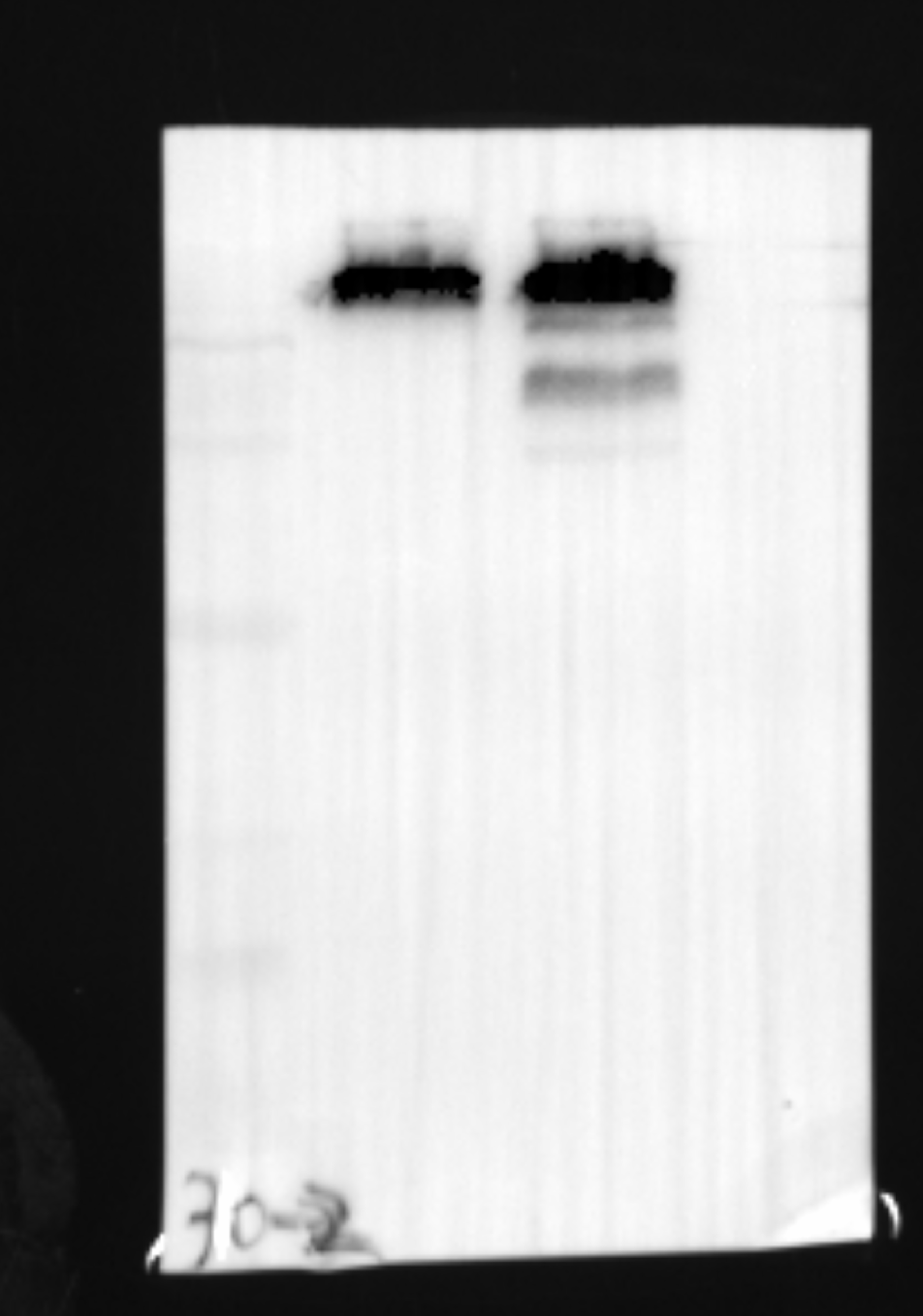

Supplement: Figure 8—source data 1. [file elife-94288-fig8-data1.zip › Figure 8–source data 1/MYHC.tif]

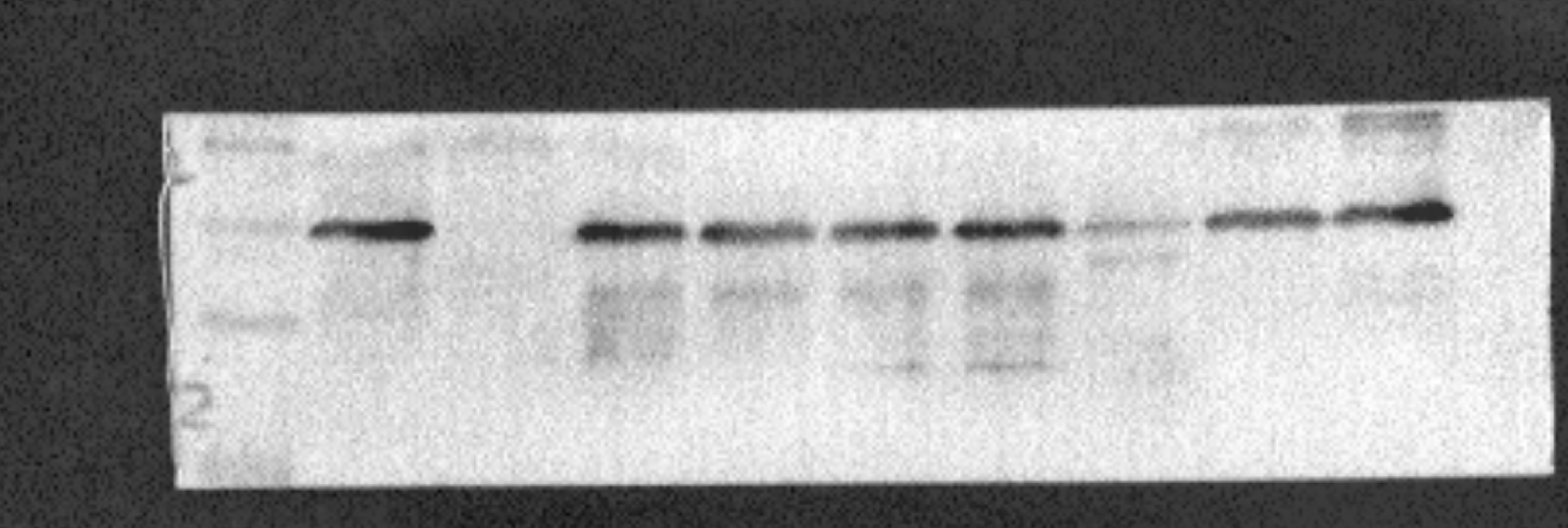

Supplement: Figure 8—source data 1. [file elife-94288-fig8-data1.zip › Figure 8–source data 1/MYOD1.tif]

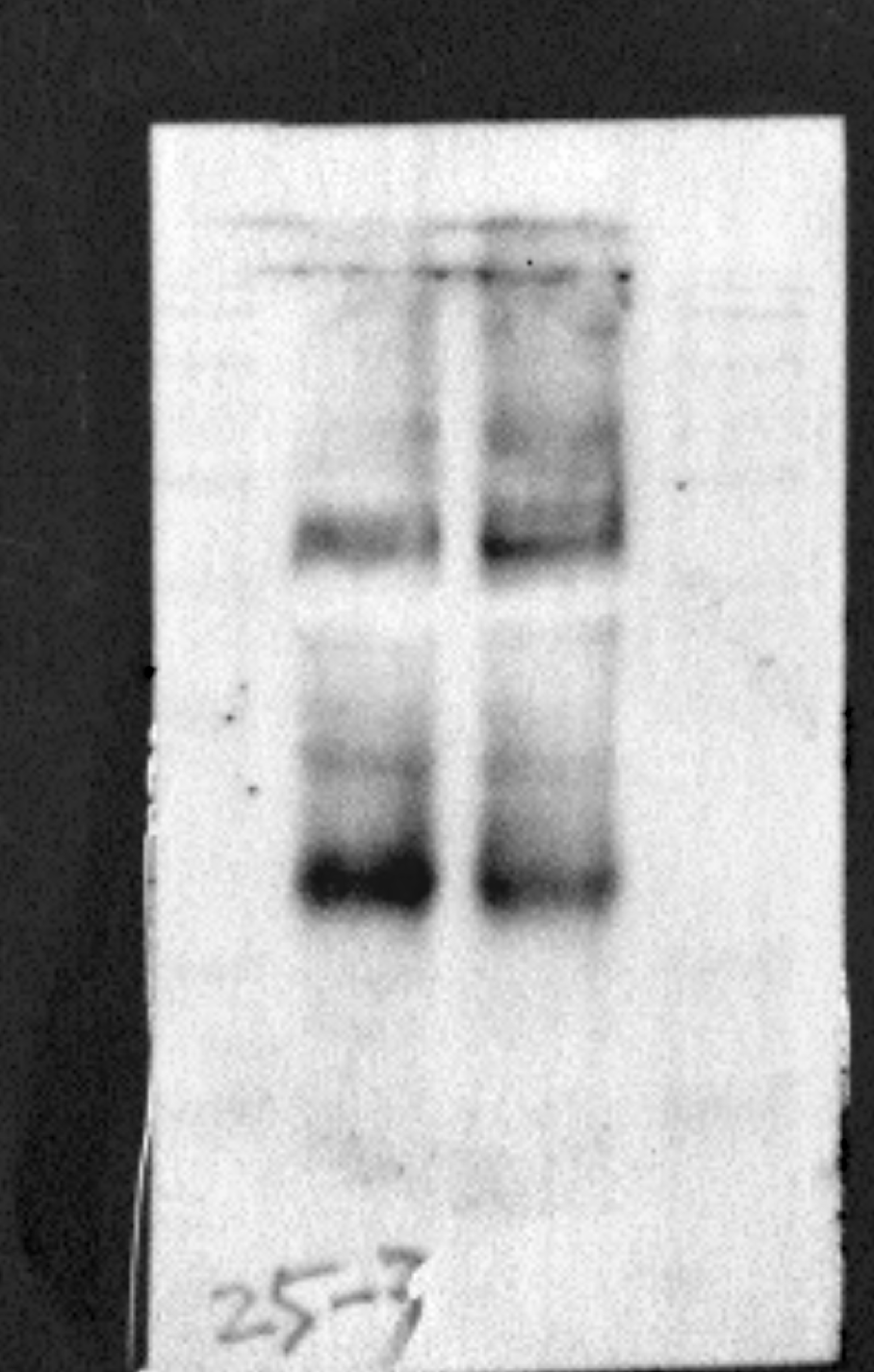

Supplement: Figure 8—source data 1. [file elife-94288-fig8-data1.zip › Figure 8–source data 1/MYOG.tif]
