# Supplementary material for: A novel mouse model for LAMA2-related muscular dystrophy with analysis of molecular pathogenesis and clinical phenotype
Source: eLife. 2025 Sep 17;13:RP94288. doi: 10.7554/eLife.94288 (PMC12443477; doi:10.7554/eLife.94288)
Supplement: Figure 1—source data 2. [file elife-94288-fig1-data2.zip › Figure 1–source data 2/Figure 1–source data 2.pdf]

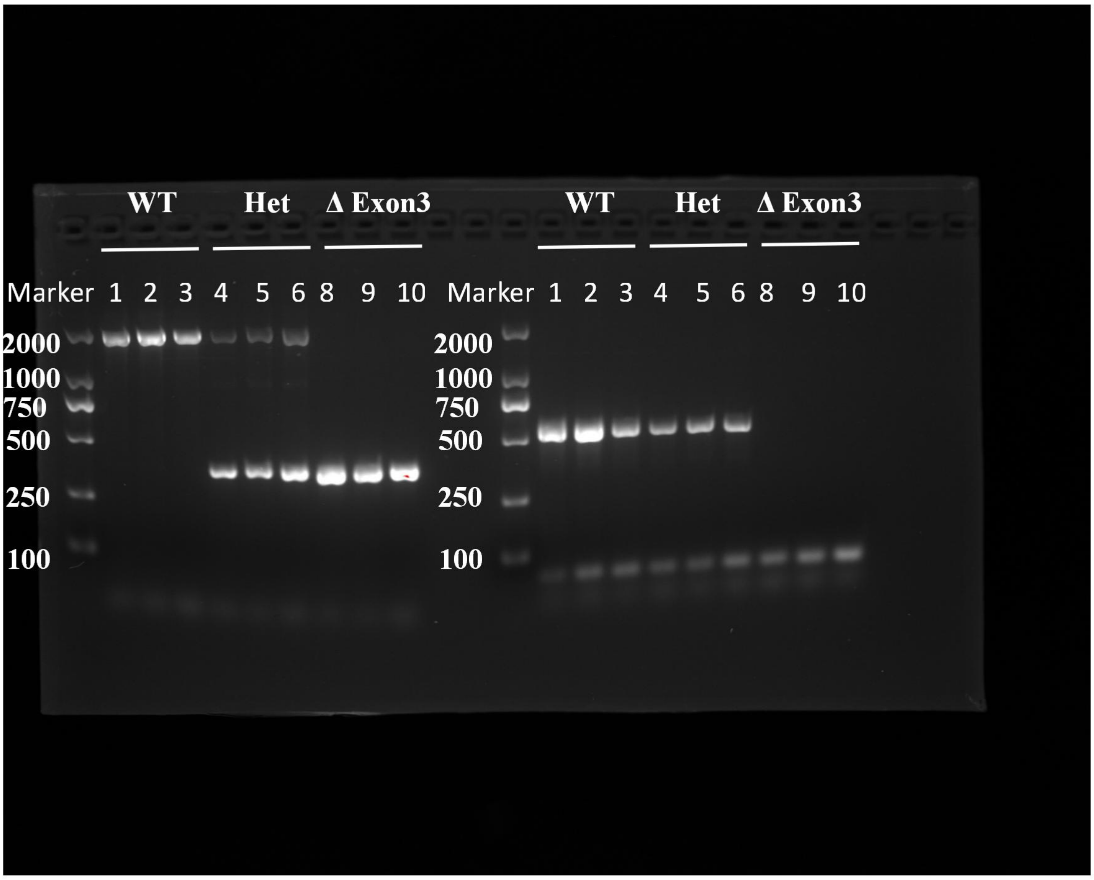

Figure 1, Source Data 2. Original membrane corresponding to Figure 1, panel B. The left part of the membrane correspond to the fragments of PCR products in PCR1, The right part of the membrane correspond to the fragments of PCR products in PCR2. Lanes with our internal codes correspond to $dy^H/+$ (Het) (lanes 1, 2, 3), $dy^H/+$ (Het) (lanes 4, 5, 6), and homozygote knockout (KO), ΔExon 3 (lanes 7, 8, 9). DNA molecular markers were employed.
