# Supplementary material for: A novel mouse model for LAMA2-related muscular dystrophy with analysis of molecular pathogenesis and clinical phenotype
Source: eLife. 2025 Sep 17;13:RP94288. doi: 10.7554/eLife.94288 (PMC12443477; doi:10.7554/eLife.94288)
Supplement: Figure 4—source data 2. [file elife-94288-fig4-data2.zip › Figure 4–source data 2/Figure 4–source data 2.pdf]

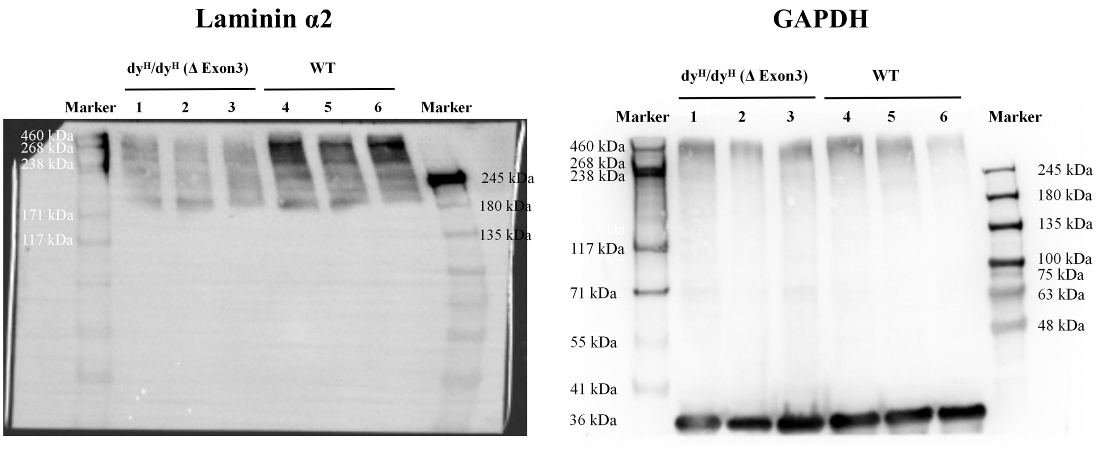

Figure 4, Source Data 2. Original membranes corresponding to Figure 4, panel B. The left membrane correspond to laminin α2 chain, The right membrane correspond to GAPDH. Lanes with our internal codes correspond to homozygote knockout (KO), dy^H/dy^H (ΔExon 3) (lanes 1, 2, 3), and $dy^H/+$ (Het) (lanes 4, 5, 6. Rainbow molecular weight markers were employed.
