# Supplementary material for: A novel mouse model for LAMA2-related muscular dystrophy with analysis of molecular pathogenesis and clinical phenotype
Source: eLife. 2025 Sep 17;13:RP94288. doi: 10.7554/eLife.94288 (PMC12443477; doi:10.7554/eLife.94288)
Supplement: Figure 8—source data 2. [file elife-94288-fig8-data2.zip › Figure 8–source data 2/Figure 8–source data 2.pdf]

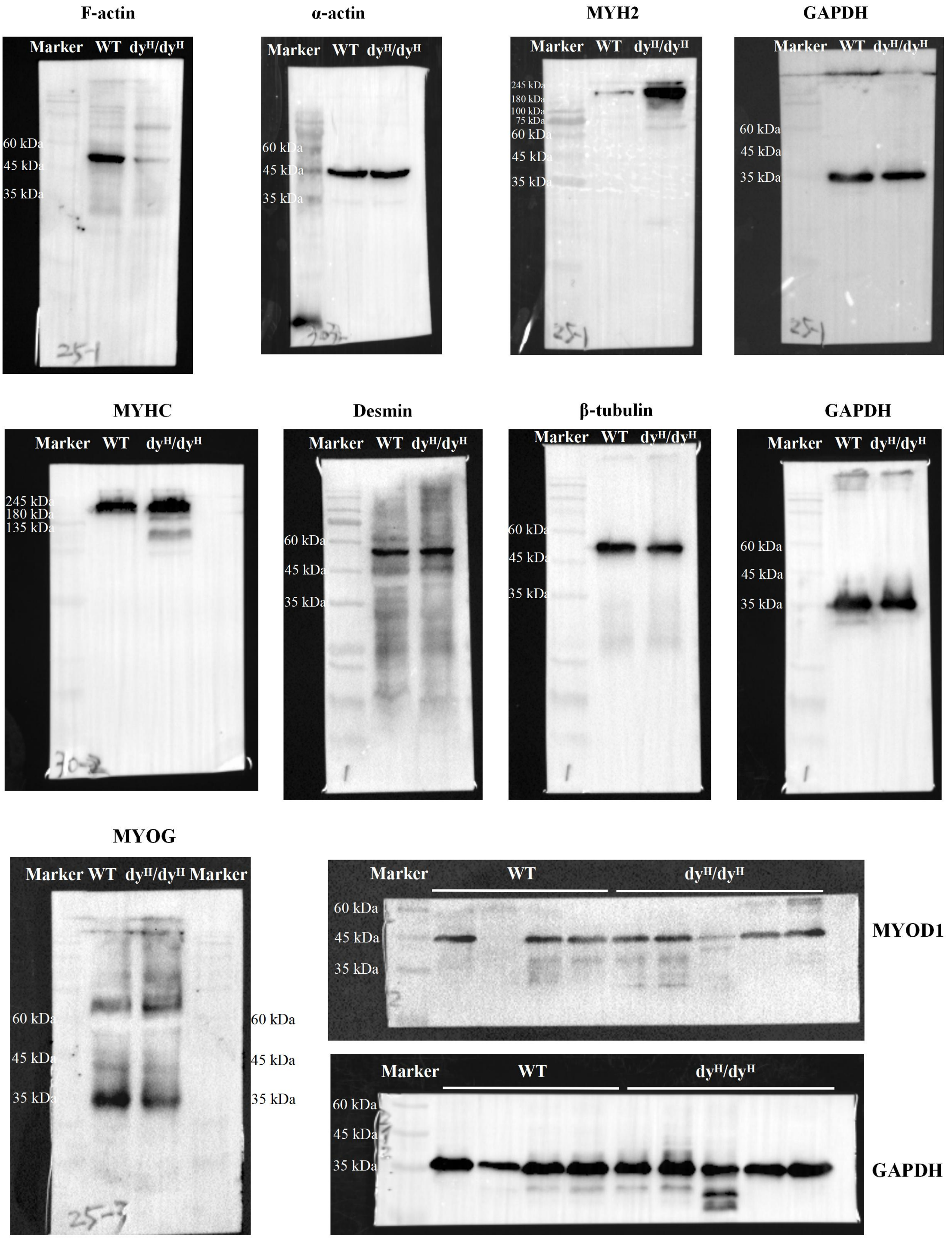

Figure 8, Source Data 2. Original membranes corresponding to Figure 8, panel B. The membranes correspond to F-actin, α-actin, MYH2, MYHC, desmin, β-tubulin, MYOG, MYOD1, and GAPDH, respectively. Lanes correspond to dy^H/dy^H and WT. Rainbow molecular weight markers were employed.
