# Supplementary material for: A novel mouse model for LAMA2-related muscular dystrophy with analysis of molecular pathogenesis and clinical phenotype
Source: eLife. 2025 Sep 17;13:RP94288. doi: 10.7554/eLife.94288 (PMC12443477; doi:10.7554/eLife.94288)
Supplement: Supplementary file 3. [file elife-94288-supp3.docx]

**Supplementary File 3.** **Comparison between the dy^H^/dy^H^ mouse with other *Lama2* deficient mice.**

| **Mouse** | ***Lama2* mutaion** | **Laminin α2 expression** | **Muscular dystrophy** | **BBB Deﬁcits** | **Life expectancy** | **References** |
| --- | --- | --- | --- | --- | --- | --- |
| dy^H^/dy^H^ | Knock-out by out-frame deletion of the exon 3 | Complete deﬁciency | Very severe | BBB dysfunction with laminin α2 deﬁciency | 3 weeks of age | This paper |
| dy/dy | Spontaneous, unknown | Reduced expression of normal sized laminin α2 | Moderate | Unknown | Before 6 months of age | *Xu et al., 1994;*  *Michelson et al., 1995* |
| dy^2J^/dy^2J^ | Spontaneous splice site mutation resulting in an in-frame deletion in the exon 2 | Slightly reduced expression of truncated laminin α2 | Mild | Unknown | After 6 months of age | *Sunada et al., 1995* |
| dy^3k^/dy^3k^ | Knock-out by inserting a reverse Neo element in the 3' end of exon 4 | Complete deﬁciency | Very severe | BBB dysfunction and increased permeability | 3 weeks of age | *Miyagoe et al., 1997;*  Gawlik *et al., 2019;*  *Menezes et al., 2014* |
| dy^W^/dy^W^ | Knock-out by inserting lacZ-neo element in the exon 1 | Severely reduced expression of truncated laminin α2 | Severe | Unknown | 5–12 weeks of age | *Kuang et al., 1998* |

*Abbreviations:* BBB, blood-brain barrier.

References

1. Gawlik KI, Körner Z, Oliveira BM, Durbeej M. 2019. Early skeletal muscle pathology and disease progress in the dy(3K)/dy(3K) mouse model of congenital muscular dystrophy with laminin alpha2 chain-deficiency. *Sci Rep* 9:14324. DOI: <https://doi.org/>10.1038/s41598-019-50550-0.PMID: 31586140
2. Kuang W, Xu H, Vachon PH, Liu L, Loechel F, Wewer UM, Engvall E. 1998. [Merosin-deficient congenital muscular dystrophy. Partial genetic correction in two mouse models.](https://pubmed.ncbi.nlm.nih.gov/9710454/) J Clin Invest 102:844-552. DOI: <https://doi.org/>10.1172/JCI3705. PMID: 9710454
3. Menezes MJ, McClenahan FK, Leiton CV, Aranmolate A, Shan X, Colognato H. 2014. The extracellular matrix protein laminin alpha2 regulates the maturation and function of the blood-brain barrier. *J Neurosci* 34:15260-15280. DOI: <https://doi.org/>10.1523/JNEUROSCI.3678-13.2014. PMID: 25392494
4. Michelson AM, [Russell](https://pubmed.ncbi.nlm.nih.gov/?size=20&term=Russell+ES&cauthor_id=16589799) ES, [PJ Harman](https://pubmed.ncbi.nlm.nih.gov/?size=20&term=Harman+PJ&cauthor_id=16589799) PJ. 1995. [Dystrophia Muscularis: A HEREDITARY PRIMARY MYOPATHY IN THE HOUSE MOUSE.](https://pubmed.ncbi.nlm.nih.gov/16589799/) Proc Natl Acad Sci U S A 41:1079-1084. DOI: <https://doi.org/>10.1073/pnas.41.12.1079.1955. PMID: 16589799
5. [Miyagoe Y, Hanaoka K, Nonaka I, Hayasaka M, Nabeshima Y, Arahata K, Nabeshima Y, Takeda S. 1997. Laminin alpha2 chain-null mutant mice by targeted disruption of the Lama2 gene: a new model of merosin (laminin 2)-deficient congenital muscular dystrophy.](https://pubmed.ncbi.nlm.nih.gov/9326364/) FEBS Lett 415:33-39. DOI: <https://doi.org/>10.1016/s0014-5793(97)01007-7. PMID: 9326364
6. Sunada Y, Bernier SM, Utani A, Yamada Y, Campbell KP. 1995. [Identification of a novel mutant transcript of laminin alpha 2 chain gene responsible for muscular dystrophy and dysmyelination in dy2J mice.](https://pubmed.ncbi.nlm.nih.gov/7655459/) Hum Mol Gene 4:1055-1061. DOI: <https://doi.org/>10.1093/hmg/4.6.1055. PMID: 7655459
7. Xu H, Christmas P, Wu XR, Wewer UM, Engvall E. 1994. [Defective muscle basement membrane and lack of M-laminin in the dystrophic dy/dy mouse.](https://pubmed.ncbi.nlm.nih.gov/8202529/) Proc Natl Acad Sci U S A 91:5572-5576. DOI: <https://doi.org/>10.1073/pnas.91.12.5572. PMID: 8202529
