# Supplementary material for: A novel mouse model for LAMA2-related muscular dystrophy with analysis of molecular pathogenesis and clinical phenotype
Source: eLife. 2025 Sep 17;13:RP94288. doi: 10.7554/eLife.94288 (PMC12443477; doi:10.7554/eLife.94288)
Supplement: Supplementary file 4. [file elife-94288-supp4.docx]

**Supplementary File 4.** **Primers of PCR-amplifications for Genotype identification.**

| **Primers** | **Primers’ sequence (5’-3’)** | **Lengths of PCR products (bp)** |
| --- | --- | --- |
| *Lama2*-F | ACTGAACCCAGGCTCCCTTTGAATC | wild-type: 571  mutant-type: 0 |
| *Lama2*-R1 | ATTAGACATCGAACCACCTCTGTTTTCA |  |
| *Lama2*-F | ACTGAACCCAGGCTCCCTTTGAATC | wild-type: 1999  mutant-type: 374 |
| *Lama2*-R2 | AACCTCAAGGCTGACACCCTGCTAA |  |

*Abbreviations:* PCR, polymerase chain reaction.
