# Supplementary material for: A novel mouse model for LAMA2-related muscular dystrophy with analysis of molecular pathogenesis and clinical phenotype
Source: eLife. 2025 Sep 17;13:RP94288. doi: 10.7554/eLife.94288 (PMC12443477; doi:10.7554/eLife.94288)
Supplement: Supplementary file 5. [file elife-94288-supp5.docx]

**Supplementary File 5.** **MRI examination imaging sequences.**

| **Series description** | **Plane** | **TR (ms)** | **TE (ms)** | **FOV (mm)** | **Slice thickness (mm)** | **Distance factor (%)** | **Matrix** | **BW (Hz/pixel)** |
| --- | --- | --- | --- | --- | --- | --- | --- | --- |
| T2-weithted (TSE) | Coronal | 3000 | 78 | 35 | 1 | 10 | 256×256 | 174 |
| T1-weighted (SE) | Coronal | 250 | 16 | 35 | 1 | 10 | 256×256 | 120 |

*Abbreviations:* TR, time of repetition; TE, time of echo; FOV, field of view; BW, bandwidth; TSE, turbo spin echo; SE, spin echo.
